# Biomarkers of Treatment Toxicity in Combined-Modality Cancer Therapies with Radiation and Systemic Drugs: Study Design, Multiplex Methods, Molecular Networks

**DOI:** 10.3390/ijms151222835

**Published:** 2014-12-09

**Authors:** Anne Hansen Ree, Sebastian Meltzer, Kjersti Flatmark, Svein Dueland, Erta Kalanxhi

**Affiliations:** 1 Department of Oncology, Akershus University Hospital, P.O. Box 1000, 1478 Lørenskog, Norway; E-Mails: sebastian.meltzer@medisin.uio.no (S.M.); erta.kalanxhi@medisin.uio.no (E.K.); 2 Institute of Clinical Medicine, University of Oslo, P.O. Box 1171 Blindern, 0318 Oslo, Norway; E-Mail: kjersti.flatmark@rr-research.no; 3 Department of Tumor Biology and Department of Gastroenterological Surgery, Oslo University Hospital, P.O. Box 4956 Nydalen, 0424 Oslo, Norway; 4 Department of Oncology, Oslo University Hospital, P.O. Box 4956 Nydalen, 0424 Oslo, Norway; E-Mail: svedue@ous-hf.no

**Keywords:** cancer treatment, radiotherapy, chemotherapy, targeted therapy, treatment toxicity, clinical study, biomarkers, proteomics, transcriptomics, systems analysis

## Abstract

Organ toxicity in cancer therapy is likely caused by an underlying disposition for given pathophysiological mechanisms in the individual patient. Mechanistic data on treatment toxicity at the patient level are scarce; hence, probabilistic and translational linkages among different layers of data information, all the way from cellular targets of the therapeutic exposure to tissues and ultimately the patient’s organ systems, are required. Throughout all of these layers, untoward treatment effects may be viewed as perturbations that propagate within a hierarchically structured network from one functional level to the next, at each level causing disturbances that reach a critical threshold, which ultimately are manifested as clinical adverse reactions. Advances in bioinformatics permit compilation of information across the various levels of data organization, presumably enabling integrated systems biology-based prediction of treatment safety. In view of the complexity of biological responses to cancer therapy, this communication reports on a “top-down” strategy, starting with the systematic assessment of adverse effects within a defined therapeutic context and proceeding to transcriptomic and proteomic analysis of relevant patient tissue samples and computational exploration of the resulting data, with the ultimate aim of utilizing information from functional connectivity networks in evaluation of patient safety in multimodal cancer therapy.

## 1. Introduction

### 1.1. Combined-Modality Radiotherapy

Radiation remains one of the most effective treatment modalities in cancer and has a central role in controlling localized disease and in palliating symptoms when cure is no longer possible. The principal therapeutic intent of exposing tumor cells to ionizing radiation is to produce irreversible DNA damage that will cause tumor cell death. In principle, if the radiation dose is high enough, all clonogenic tumor cells in the target volume will be exterminated, and the therapy is curative. Technological advances in radiation delivery have enabled development of physical high-precision treatment protocols to improve patient tolerability for dose escalation. Yet, radiotherapy is fundamentally a biological intervention [1], and further technological refinements may not necessarily lead to a tangible improvement in cancer management.

In the past decade, the benefit of chemoradiotherapy (CRT), *i.e.*, the addition of concomitant cytotoxic agents to radiotherapy, has been demonstrated for a range of tumor types. One example is locally advanced rectal cancer (LARC), which comprises primary tumors that infiltrate beyond the rectal wall to an extent that precludes primary surgical removal with sufficient microscopic margins. Randomized studies have highlighted the central role of neoadjuvant CRT in macroscopic down-sizing and control of subclinical tumor manifestations within the pelvic cavity, to enable resection of the residual tumor within its entire extension for the ultimate improvement of outcome [2]. Yet, there is compelling evidence from large cohorts of LARC patients given neoadjuvant CRT that long-term survival benefit is contingent on considerable or factual complete tumor response [3], supporting the notion that eradication of tumor clonogens is essential for favorable therapeutic results.

Within this frame of reference, and with recent insights into molecular radiobiology, there is an increasing opportunity for rational integration of molecularly targeted therapeutics in clinical radiotherapy in an effort to optimize radiation effects [4]. Recognizing that biological therapies frequently have modest single-agent activities, they may rather have potential both to amplify the cytotoxicity elicited by radiation-induced DNA damage and to counteract the resulting activation of intracellular signaling defense responses. The landmark study confirming major improvement in post-radiotherapy survival outcome for head-and-neck cancer patients concomitantly treated with cetuximab, an anti-EGFR antibody [5], was the first substantial proof of this concept.

### 1.2. Treatment Toxicity

The unrivalled efficacy of radiation in treatment of local tumor manifestations is a reflection of a delivered radiation dose that is commonly at the limit of normal tissue tolerance, and any adverse event that causes an interruption in the radiation delivery is likely to have a negative impact on the probability of tumor control. Importantly, in combining a systemic drug with radiotherapy, synergistic toxicity profiles may prevail [4]. It is therefore acknowledged that the achievements in survival outcome resulting from the more efficacious therapies that include increasingly complex multimodality programs are at the price of extended limits of treatment intensity and patient tolerance.

In contrast to studies that examine effect and toxicities of single-agent treatment, the combination of a systemic compound with radiation is a more complex trial context that demands special consideration of study design and endpoints that reflect both radiation effect and potential independent and overlapping toxicities of the two modalities. This requires particular attention on the definition of patient eligibility and radiation dose-volume relationships in evaluating normal tissue toxicities. For example, while disease location is less critical for the evaluation of treatment tolerability in systemic therapy protocols, in radiotherapy the anatomical site of the target lesions determines the adjacent organs at risk. Hence, to enable interpretation of toxicity data of combined-modality therapies, the anatomical disease site being irradiated needs to be clearly specified in the protocol [4], as more broadly discussed in Section 3.

In pelvic curative radiotherapy, the development of acute intestinal toxicity, clinically presenting as significant diarrhea and associated metabolic disturbances, represents the major limitation to delivering the intended radiation dose. Hence, radiation-induced acute enteritis is strongly associated with interruption or premature cessation of treatment and as a result, an adverse patient outcome [6]. Radiation-induced early toxicity is commonly experienced either towards the end of the therapy course or within a few weeks of treatment completion, typically in normal tissues with a hierarchical proliferative structure, such as the mucosal linings of the gastrointestinal tract. Such side effects are transient in nature, but emerging data also indicates that severe early toxicity may be causally related to long-term sequelae in patients completing curative radiotherapy [7].

### 1.3. Exploring Toxicity Mechanisms—A “Top-Down” Strategy

**Figure 1:**
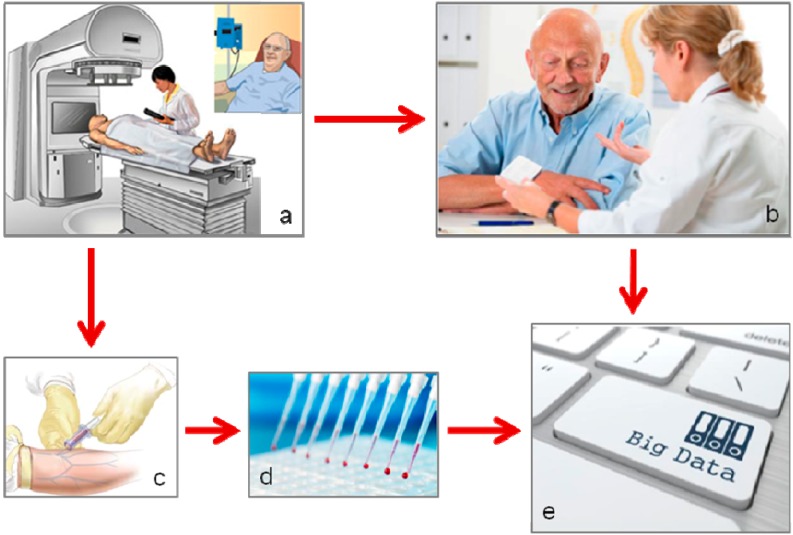
Pathway of study conduct in molecular portraying of clinical toxicity profiles. (**a**) Patient treatment within a prospective study; (**b**) Assessment of treatment toxicities; (**c**) Sampling of relevant normal tissue; (**d**) Multiplex analysis of collected samples; (**e**) Computational data analysis. Images are purchased [8] or adapted with permission [9].

Normal tissue toxicity in cancer therapy, as seen in trials and common clinical practice, is most likely a deterministic variability among individuals, *i.e.*, caused by underlying disposition for given pathophysiological mechanisms. In view of the complexity of biological responses to injury from combined-modality radiotherapy regimens, a “top-down” strategy, starting with the systematic assessment of side effects within the context of prospective therapy studies and attempting to unravel putative links with predisposing factors in the affected individuals, provides a research avenue that has become increasingly appealing with the advent of multiplex high-throughput laboratory technologies and computational tools (Figure 1).

The present communication will discuss our experiences from two biomarker studies of combined-modality pelvic radiotherapy. In these prospective studies, we applied a robust and validated evaluation tool for treatment-related adverse events. The study patients had consented to the sampling of normal tissues that were relevant for exploration of biomarkers of the anticipated treatment toxicity. Following the ongoing analysis of the patient samples with two different multiplex technologies, the resulting datasets will be explored through various bioinformatics algorithms. Conceptually, this pathway of clinical study conduct proceeding to molecular portraying of toxicity profiles using appropriate software analysis tools may form a template for how to assess and evaluate endpoints of patient safety in contemporary multimodal cancer therapy.

## 2. Biomarkers of Treatment Toxicity—Study Conduct

### 2.1. The Locally Advanced Rectal Cancer—Radiation Response Prediction (LARC-RRP) Study

The LARC-RRP study [10] was conducted in the context of curative treatment of LARC with the notion that a second cytotoxic drug as an additional component to the established neoadjuvant fluorouracil-based regimen might improve the rate of complete tumor response. This phase II study for neoadjuvant therapy followed by surgery and no further treatment enrolled patients from October 2005 to March 2010 and has at present a follow-up time of five years for almost all of the study patients. The treatment protocol consisted of two cycles of the Nordic FLOX chemotherapy regimen [11] followed by CRT, in which radiation was prescribed to 50 Gy in 2-Gy daily fractions with concomitant capecitabine and oxaliplatin chemotherapy [12]. Surgery was planned 6–8 weeks after completion of the neoadjuvant treatment.

**Figure 2:**
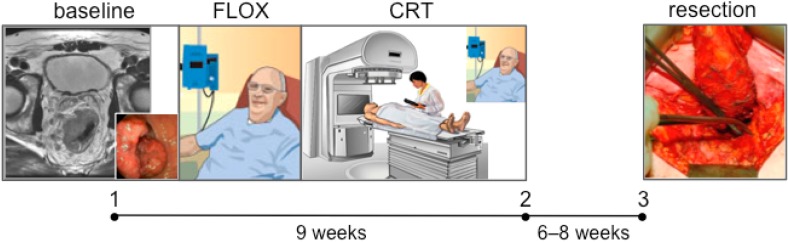
*Locally Advanced Rectal Cancer—Radiation Response Prediction:* design of the circulating biomarker study. Serum samples (denoted by closed circles) were collected at baseline (*i.e.*, the time of diagnosis with rectal endoscopy and magnetic resonance imaging of the pelvic cavity; 1), following FLOX chemotherapy and chemoradiotherapy (CRT; 2), and before resection of the residual primary tumor (3). Treatment toxicity, assessed as detailed in Section 3, was recorded immediately following the completion of neoadjuvant treatment and repeatedly before the surgery. Images are from own work or adapted with permission [9,13].

The study has generated an extensive biobank and imaging databank, consisting of tumor biopsies, surgical specimens, bone marrow and serial blood samples, functional imaging readouts, and radiation treatment-planning histograms, as well as a comprehensive clinical database on a range of disease outcome parameters (response and toxicity data). Specifically to quest circulating biomarkers of treatment toxicity, multiplex analysis of cytokine profiles in the prospectively archived serial serum samples collected during the neoadjuvant treatment course (Figure 2) is currently undertaken, as outlined below.

### 2.2. The Pelvic Radiation and Vorinostat (PRAVO) Study

Within our preclinical program, histone deacetylase (HDAC) inhibitors, targeting tumor histone acetylation, have been systematically investigated as radiosensitizing drugs. Following the initial demonstration that HDAC inhibitors enhanced radiation-induced clonogenic suppression of experimental human colorectal carcinoma (CRC) cells in culture [14,15], human CRC xenograft models showed significantly prolonged tumor growth delay to fractionated radiation combined with daily treatment with the HDAC inhibitor vorinostat compared to that seen with radiation treatment alone [16]. Furthermore, tumor volume shrinkage of irradiated hypoxic xenografts in mice given vorinostat was similar to the xenograft growth retardation resulting from irradiation under normoxic conditions, demonstrating that the drug had reversed the radiation-resistant hypoxic phenotype [17].

As LARC comprises heterogeneous pelvic cavity tumors with predominant hypoxic components, refinement of the treatment protocol with a biologically targeted drug with radiosensitizing properties might improve local tumor control. However, recognizing that treatment toxicities which may cause interruption in the radiation delivery are likely to have a negative impact on the probability of tumor control, only patients that are not candidates for any curative radiotherapy protocol should be regarded as eligible in a trial evaluating tolerability of a first-in-human combination of radiation with a targeted drug [4].

**Figure 3:**
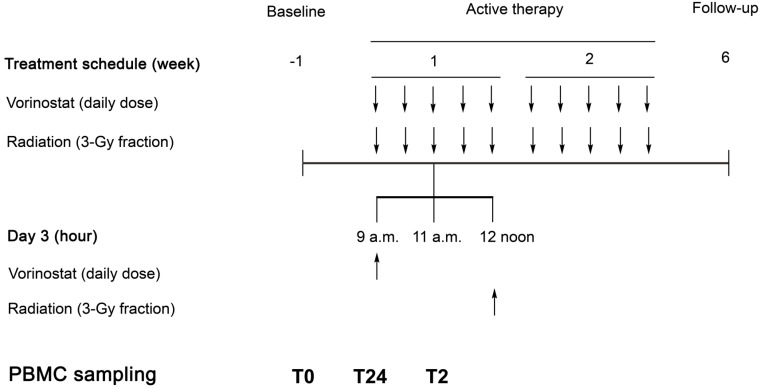
*Pelvic Radiation and Vorinostat:* design of the vorinostat biomarker study. Arrows indicate administration of therapy (daily vorinostat dose at 9 a.m. and daily exposure to a 3-Gy radiation dose at 12 noon) for ten days. Peripheral blood mononuclear cells (PBMC) were sampled at baseline, before commencement of treatment (T0), and on active therapy, two hours (T2; at 11 a.m. on day 3) and 24 h (T24; at 9 a.m. on day 3) after the previous dose of vorinostat. Treatment toxicity, assessed as detailed in Section 3, was recorded continuously during treatment and re-examined six weeks after treatment completion (at follow-up). The figure has been published previously by Ree and co-workers and is reproduced with permission [22].

In the PRAVO phase I study [18] for symptom palliation in advanced bowel carcinoma, sequential patient cohorts were exposed to escalating dose levels of vorinostat combined with palliative radiotherapy to pelvic target volumes. This study was the first to report on the therapeutic use of an HDAC inhibitor in clinical radiotherapy [19], and was designed to meet several endpoints, such as the demonstration that vorinostat reached the specific target (detection of tumor histone acetylation) and the applicability of non-invasive tumor response assessment (using functional imaging). Because common side effects of vorinostat single-agent therapy include intestinal toxicities [20], the primary objective of the study was to determine tolerability in combination with the pelvic radiation [19,21]. Importantly, molecular biomarkers of vorinostat action in this combined-modality context are being explored in study patients’ peripheral blood mononuclear cells (PBMC), representing drug-exposed normal tissue (Figure 3). Initially, we have observed that biomarkers of vorinostat activity reflect the appropriate timing of drug administration in the fractionated radiotherapy protocol [22]. We are currently examining biomarkers of vorinostat-induced toxicity, as outlined below.

## 3. Evaluation of Treatment Toxicity—Clinical Assessment

### 3.1. Common Terminology Criteria for Adverse Events (CTCAE)

It is contended that quantification of treatment toxicity inherently is much more complex than quantification of treatment efficacy because of the huge variation in severity of adverse events among individuals treated for cancer. However, the National Cancer Institute’s CTCAE [23] were established as a system for recording toxic effects with all types of cancer therapy and to uniform severity scaling. Close attention was paid to the boundary between grade 2 and grade 3, demarcating a clearly higher level of severity [24].

In general, CTCAE grade 1 toxicities are findings of negligible impact on activities of daily life, CTCAE grade 2 toxicities represent moderate adverse events, and CTCAE grade 3 and 4 toxicities reflect injury of grave or life-threatening severity, respectively. This implicates, in addition, that grade 3–4 events are often used to trigger dose reductions or other therapy adjustments in addition to intensified supportive care intervention, which usually involves hospital admission.

The LARC-RRP and PRAVO studies had prospective design; thus, toxicity was recorded prospectively and uniformly, according to CTCAE. This is of utmost importance as toxicity score was the hard endpoint in both of the studies. In general, our understanding of underlying mechanisms of treatment toxicity lags far behind that of tumor response [25], a realization that strengthens the necessity of applying validated scientific methodologies at every step of the assessment and tentative biological understanding of normal tissue response to treatment exposure. 

### 3.2. Intestinal Toxicity in Pelvic Radiotherapy

Among the strongest determinants of normal tissue toxicity in radiotherapy are the size of the radiation target volume and the radiation dose distribution within this volume [6,26]. When the radiotherapy is delivered to appropriate target volumes as determined by state-of-the-art imaging-based treatment planning, as was the case in both of the LARC-RRP and PRAVO studies, the extent of involved small bowel in the therapeutic target volume and dose-volume histograms for any other exposed normal tissues can be retrieved from the patients’ individual treatment-planning data-sets. By this, the normal tissue dose-volume effects can be quantified and enable the estimation of their contribution to treatment-induced adverse events. This aspect in the evaluation of treatment tolerability is particularly important in studies of therapy intensification, such as radiation dose escalation, the possible enhancement effect of the radiation-drug scheduling, or the addition of radiosensitizing drugs.

### 3.3. LARC-RRP and PRAVO—Clinical Toxicity Profiles

In studies that are designed as investigations into the safety of combining a radiosensitizing drug with pelvic radiotherapy, in which acute bowel toxicity is frequently encountered by the radiation exposure alone, it may be difficult to decide whether or not an adverse event occurring during treatment is greater than might be expected for either of the therapeutic components. It is particularly challenging to evaluate the contribution of the systemic component to the overall treatment toxicity if its separate toxicity profile is indistinguishable from that of the radiotherapy, and to determine whether a CTCAE grade 3–4 event should be considered as caused by the systemic agent.

#### 3.3.1. Curative Combined-Modality Therapy

In an intensified curative radiation schedule at the limits of normal tissue tolerance, the increased risk of interruption or premature cessation of the treatment and hence, deleterious effects on patient outcome, must be specifically addressed in the study design. In order to meet this challenge in the LARC-RRP study, the neoadjuvant CRT schedule was continuously adjusted according to toxicity by reducing doses of or entirely discontinuing oxaliplatin, capecitabine, or radiotherapy in that order of priority [12], reflecting the relative importance of the three therapeutic components within the combined-modality treatment regimen.

As shown by Table 1, severe treatment-induced diarrhea (*i.e.*, CTCAE grade 3) was reported towards the end of the neoadjuvant treatment course by 10% of study patients; however, the majority of events had been resolved at the time the patients were evaluated before surgery, which took place 4–6 weeks after CRT completion. This rather low incidence of CTCAE grade 3 adverse events in a curative treatment setting reflects the precaution criteria inherent in the study protocol. Yet, CTCAE grade 3 diarrhea implicates an interruption of the principal component of the neoadjuvant therapy—the course of daily radiation delivery. As a specific consequence, which was not foreseen when the study protocol was designed, serum was not sampled at CRT completion for three out of the eight patients that reported CTCAE grade 3 diarrhea. This finding may reflect the institutional standard of recommencement of curative radiotherapy as soon as the specific toxicity was resolved, which possibly resulted in de-synchronization of the time of treatment completion and the study-specified once-weekly timing of serum sampling and hence, the inadvertent omission of the latter. The reason why a significant number of the study patients did not receive the protocol-specified toxicity assessments (8 individuals) at evaluation is not specifically known.

**Table 1:** *Locally Advanced Rectal Cancer—Radiation Response Prediction:* the number of study patients (in whom multiplex cytokine profiling of serial serum samples was undertaken; *n* = 80) reporting diarrhea, graded according to the Common Terminology Criteria for Adverse Events (CTCAE), at the time of diagnosis (baseline), following the course of neoadjuvant treatment with the Nordic FLOX regimen and chemoradiotherapy (CRT), and at evaluation prior to tumor surgery.

CTCAE Scoring	At Baseline	At Completion of FLOX + CRT	At Evaluation
CTCAE grade 0 diarrhea	66	36	53
CTCAE grade 1 diarrhea	10	22	17
CTCAE grade 2 diarrhea	4	14	0
CTCAE grade 3 diarrhea	0	8	2
CTCAE grade 4 diarrhea	0	0	0
not determined	0	0	8

#### 3.3.2. Non-Curative Combined-Modality Therapy

In a study setting of evaluating tolerability of a first-in-human combination of radiation with a targeted therapeutic, only patients that are not candidates for any curative radiotherapy protocol should be regarded as eligible. As a general principle, committees for medical and health research ethics will waive approval of first-in-human experimental therapeutic approaches in patients with curative intention of standard treatment.

The PRAVO study adopted the conventional 3 + 3 expansion cohort design [27], which involves treating cohorts of patients with gradually increasing doses of the investigational agent. Dose escalation takes place for every third evaluable patient that has completed the preceding level. An individual patient cohort is expanded up to six if one of the three initial patients experiences a dose-limiting toxicity (DLT). If two patients at a given dose level experience a DLT, the maximum-tolerated dose is determined as the preceding dose level, provided an observed incidence of DLT in no more than one of the six patients.

Hence, the PRAVO study was undertaken in sequential patient cohorts exposed to escalating dose levels of vorinostat (level 1–4) given three hours before the daily pelvic palliative radiotherapy fraction. As shown by Table 2, the reported CTCAE grade 3 toxicities (*i.e.*, DLTs) were intestinal and related adverse events. One patient at vorinostat dose-level 3 and two individuals at the dose-level 4 experienced grade 3 adverse events.

**Table 2:** *Pelvic Radiation and Vorinostat:* individual study patient’s age, vorinostat dose level, relative small bowel volume receiving the fully prescribed radiation dose of 30 Gy (SBV 30), and reported Common Terminology Criteria for Adverse Events (CTCAE) grade 3 intestinal toxicities.

Age of Study Patient (years)	Vorinostat Dose Level	SBV 30 (%)	CTCAE Grade 3 Adverse Events
77	1	0	
49	2	11	
66	2	3	
87	3	40	anorexia, fatigue
47	3	14	
77	3	1	
82	3	0	
81	3	0	
55	4	6	
83	4	3	diarrhea, anorexia, hyponatremia
85	4	0	
75	4	0	diarrhea, fatigue, hypokalemia
62	4	0	
45	4	0	

Fourteen study patients had radiation treatment-planning imaging scans visualizing the entire abdominal and pelvic cavities. All individual loops of the small bowel could therefore be contoured on the scans, enabling the generation of total small bowel dose-volume histograms [6]. Hence, for each of these 14 study patients, the relative small bowel volume receiving the fully prescribed radiation dose of 30 Gy could be quantified.

As indicated by the Table 2 data, the single patient reporting CTCAE grade 3 toxicities (anorexia and fatigue) at the vorinostat dose-level 3 may have experienced an adverse radiation dose-volume effect rather than a toxic effect of the investigational drug since she had 40% of her total small bowel volume exposed to the fully prescribed radiation dose of 30 Gy. In all of the other study patients, the relative volumes of small bowel receiving such a high radiation dose were substantially smaller. The two patients reporting CTCAE grade 3 toxicities at the highest dose level of vorinostat (dose-level 4) had radiation dose-volume records that essentially were indistinguishable from the estimates in patients without any treatment-related grade 3 adverse events. Hence, in the PRAVO study, there seemed to be a threshold volume of irradiated small bowel that distinguished between high-risk and low-risk patients with respect to severe bowel toxicity. This has also been proposed previously as a biomarker of treatment toxicity in pelvic radiotherapy [28,29], and does also emphasize that where overlapping toxicities between a systemic compound and radiation are anticipated, it is highly beneficial if detailed radiation dose-volume constraints are described within the treatment protocol [4].

## 4. Biomarkers of Treatment Toxicity—Array-Based Analyses

### 4.1. Serial Serum Samples—Cytokine Profiling

In the LARC-RRP study, we hypothesized that a serum cytokine profile specific for mucosal inflammation (*i.e.*, enteritis) may be a biomarker of intestinal treatment toxicity since acute radiation enteropathy is strongly associated with activation of mucosal inflammatory cytokines [30,31], also in experimental models [32]. Recently, we have therefore utilized a protein expression array technology to discover changes in serum cytokine profiles in the LARC-RRP study patients throughout the neoadjuvant therapy course.

Specifically, we have used the RayBio^®^ Label-based Antibody Array (RayBiotech, Inc., Norcross, GA, USA) for detection of 507 different proteins, which include cytokines, growth and angiogenic factors, adhesion molecules, and matrix metalloproteinases. The serum samples are biotinylated on the primary amine of each serum protein and then incubated onto the arrays printed with capture antibodies, followed by addition of a streptavidin-conjugated fluorescent dye, HiLytePlus™ 555 (AnaSpec, Inc., Freemont, CA, USA), for signal detection [33].

Because this protein expression technology is rather recent, several means of data quality control have been undertaken. The full number of array slides was printed in repeat batches, but variation in signal distribution seems to be independent of the different batch series. Global median normalization has been performed to compensate for the slight variation among the slide batches. A subsequent scatter plot analysis has revealed that intra-patient sample variation is significantly less than the sample variation between different individuals.

### 4.2. Serial PBMC Samples—Gene Expression Profiling

Ideally, in pinpointing the causative biological pathways that reflect treatment severity at the individual level, the normal tissue that best manifests the actual clinical phenotype should be analyzed. However, practical issues may relate to the availability and quantity of tissue retrieval and hence, whether obtained tissue samples are high-quality research material. Therefore, at the study population level, a surrogate normal tissue is commonly accepted as being more feasible for correlative mechanistic analysis.

In the PRAVO study, we hypothesized that gene expression profiles in patients’ PBMC would represent an activated gene program reflecting normal tissue responses to the HDAC inhibitor vorinostat. We therefore isolated PBMC RNA from samples collected from the study patients before commencement of treatment (T0) and on treatment two and 24 h (T2 and T24) after the patients had received vorinostat.

The array analysis was undertaken at the Norwegian Genomics Consortium (Oslo, Norway). Briefly, cRNA synthesis, amplification, and hybridization to Illumina Human WG-6 v3 Expression BeadChip arrays (Illumina, Inc., San Diego, CA, USA), containing 48,000 probes, were carried out as per manufacturer’s instructions. Signal intensities were extracted by the BeadArray Reader Software (Illumina Inc., San Diego, CA, USA), and raw data were imported into the GenomeStudio v2010.1 Software, Gene Expression module v1.6.0 (Illumina Inc., San Diego, CA, USA) [22]. Following quality control and pre-processing, the data were log_2_-transformed. The primary array data are available in the Gene Expression Omnibus data repository [34] with accession number GSE46703.

## 5. Biomarkers of Treatment Toxicity—Computational Analyses

### 5.1. Integrated Systems Biology-Based Prediction of Treatment Safety

Until recently, mechanism-based prediction of treatment toxicity has been a rather new discipline but is now in accelerating development. In the past years, rapidly evolving data resources in molecular omics (e.g., transcriptomics and proteomics) and their relationship to human physiology have been increasingly used to process the input data that are utilized in the generation of prediction models for treatment outcome. However, especially in toxicology, the available data resources have resided in fragmented forms but are now tangibly accessible as public data sources for use in systems biology-based prediction of treatment safety. A useful summary of key databases for this purpose was recently presented by Bai and Abernethy [35].

**Figure 4:**
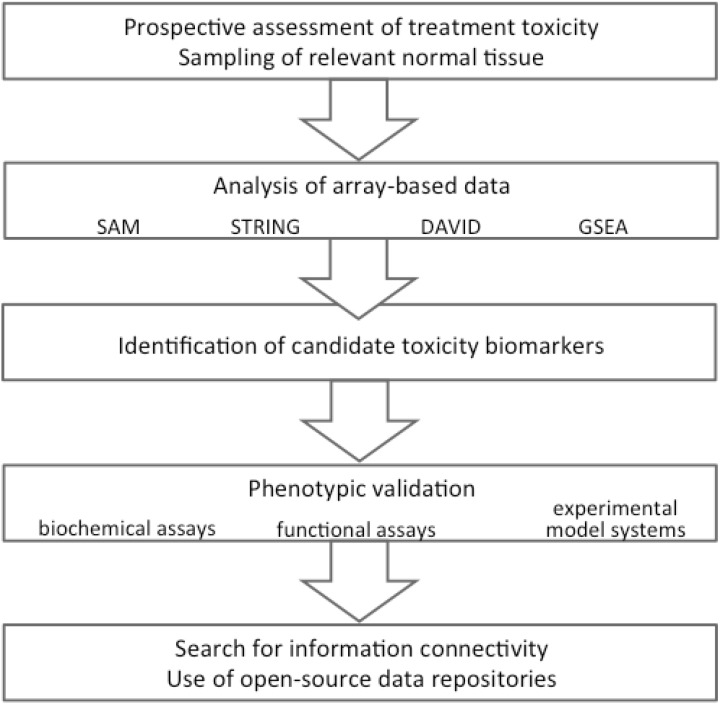
Mechanism-based prediction of treatment toxicity—an example. SAM: Significance Analysis of Microarrays; STRING: Search Tool for the Retrieval of Interacting Genes/Proteins; DAVID: Database for Annotation, Visualization and Integrated Discovery; GSEA: Gene Set Enrichment Analysis.

To date, specific databases for radiation therapy information in this particular context are scarce or even missing. One reason may be that relevant information spanning the entire path from the functional perturbation of relevant molecular targets to the treatment toxicity experienced by the patients, or *vice versa*, has not been systematically compiled. In the following, we will present how we envisage a rational application of publically available databases and open-source tools in our ongoing quest of toxicity biomarkers of genuine significance in multimodal cancer therapy (Figure 4).

### 5.2. Exploring Proteomic Signatures

As of today, protein array-analyzing software packages, such as concentration-dependent analysis (to correct for variable quantities of spotted material on the chip), are available in a limited degree compared to the abundant gene expression array counterparts. Awaiting the likely development of specific proteomic software, it has until now been common to use DNA-specific software to analyze the protein data [36]. Examples are software for reliable spot-finding on slide images, *Z*-score analysis (to determine which of the array signals are significantly different from the expected values), and Significance Analysis of Microarrays (SAM) calculations.

**Figure 5:**
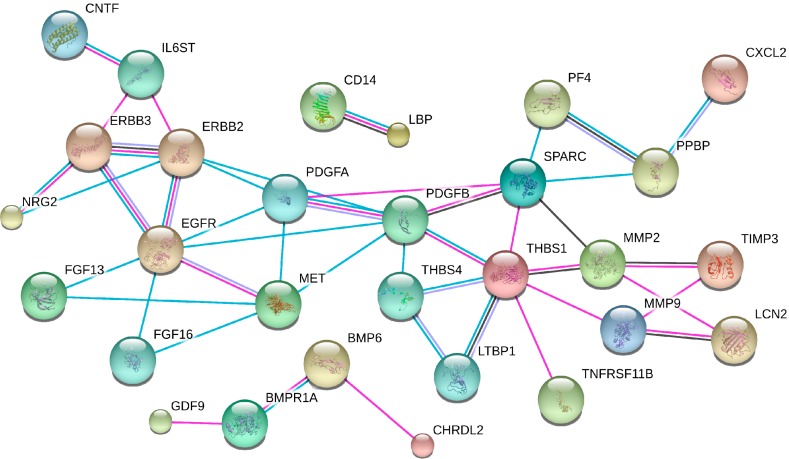
*Locally Advanced Rectal Cancer—Radiation Response Prediction:* the network of interacting proteins, shown by their gene names and whose serum levels were altered following the course of neoadjuvant treatment of the study patients, as determined by Search Tool for the Retrieval of Interacting Genes/Proteins. Connective lines represent the following types of evidence: experiments (**pink**), databases (**turquoise**), co-expression (**black**), and co-occurrence (**purple**).

In the LARC-RRP study, we use SAM [37] to identify potential toxicity biomarkers from the serum cytokine profiling. Using this open-access program, a score will be assigned to proteins based on the change in expression relative to the standard deviation of repeat measurements. This will determine false discovery rates (FDR) that compensate for the risk of detecting chance hits. Following this initial analysis, we have been interested to explore whether alterations in circulating proteins during the course of neoadjuvant treatment of study patients might reflect integrated biological processes in terms of adverse treatment effects. Functional coupling analysis using the Search Tool for the Retrieval of Interacting Genes/Proteins software has revealed a high degree of interaction between some of the significantly changed proteins (Figure 5). We envisage this approach will ultimately enable us to distinguish proteins with significant variation throughout the course of neoadjuvant therapy from the large multiplex analysis (the RayBio^®^ Label-based Antibody Array). However, as the multiplex antibody arrays are semi-quantitative, enzyme-linked immunosorbent assays will subsequently be performed directly on the study patients’ serum samples to validate the array findings.

The main advantage of using array technology for serum protein profiling in this study is the ability to analyze several factors simultaneously and therefore get a more in-depth picture of the biological processes altered in response to treatment. However, as it is common with high-throughput technologies, certain limitations should be considered. Detection of only 507 proteins out of thousands of serum factors represents an investigation bias. In addition, there are technical aspects such as protein stability and non-specific binding that may result in low detection of some proteins and generation of false positives, respectively [38].

With the emergence of individualized cancer medicine and the increasing amount and complexity of available data, there is a need for development of clinical decision support systems based on prediction models of treatment outcome [39]. Hence, we aim to develop a prediction model of treatment toxicity, combining circulating factors identified by the serum cytokine profiling and clinical markers commonly used in current practice, applying a multistage process, and correlating to the CTCAE outcome parameters. Both “simple” multivariate analysis and more advanced prediction modeling will be endeavored.

### 5.3. Exploring Transcriptomic Signatures

Single-agent vorinostat in continuous dosing is known to be tolerated at 400 mg daily, corresponding to the highest dose level (dose-level 4) of the PRAVO study, with the most common side effects being intestinal and related events [20]. Hence, when combining this radiosensitizing drug with pelvic radiation, synergistic normal tissue effects were anticipated. This calls attention to the identification of PBMC biomarkers at dose-level 4, the level where the specific vorinostat-induced DLTs were reported by study patients. Two different methods will be used to examine differential PBMC gene expression at T2 and T24, compared to T0, at each of the escalating dose levels of vorinostat, and further analyzed for whether there may be a quantitative or qualitative change in the expression at dose-level 4. The data will be analyzed in the context of expression changes in individual genes as well as in sets of genes belonging to known pathways.

Again, the SAM software will be used to analyze differential expression at the single gene level while taking into account the large number of genes in the data-set by applying the setting of paired-comparisons and an FDR rate of 5%. Over-representation of the differentially expressed genes in different biological process categories will then be quested with the Database for Annotation, Visualization and Integrated Discovery software [40,41]. The intention is to discover groups of genes that jointly govern functional phenotypes of vorinostat toxicity and subsequently validate the findings in experimental model assays with high power to predict the actual phenotypic responses.

When considering the large number of probes (approximately 48,000) in each microarray, small changes in the expression of individual genes may not be determined as significant; but again, those changes may be of biological importance when they occur in a collection of genes belonging to the same biological pathway. For this reason, an analysis method that considers all of the genes in an experiment, not only those above an arbitrary cutoff in terms of fold-change or statistical significance, is advisable. Hence, enrichment in sets of genes that are cross-correlated within the same biological processes will be investigated using the Gene Set Enrichment Analysis (GSEA) software, where genes are permuted instead of the phenotype and with an accepted FDR rate of 5% [41]. In performing the GSEA comparisons between PBMC samples, two by two, annotated gene-sets from the Molecular Signatures Database [42] will be used as per recommendation. Featuring several advantages over single-gene methods, GSEA can also be applied to serum proteomics datasets or other biological profiles of susceptibility to cancer therapy.

## 6. Prediction of Toxicity in Cancer Therapy—Integration of Multilevel Information

### 6.1. Defining Clinical Treatment Toxicity

As this communication has aimed to illustrate, a combined-modality therapy study may be designed to demonstrate a number of key questions; still, the primary objective will be to assess whether the combination of the systemic drug and radiation is safe and tolerable. Importantly, to enable full interpretation of outcome toxicity data, the disease sites being irradiated will require to be specified in the study eligibility criteria together with a description of detailed radiation dose-volume dependencies within the treatment protocol. This will also facilitate identification of adverse radiation effects that are separate from toxic effects of the systemic agent. Yet, it may frequently be challenging to evaluate the contribution of the systemic component to the overall treatment toxicity, particularly if its toxicity profile is indistinguishable from acute normal tissue effects in the radiation target volume. Therefore, the study design may include the collection of drug-exposed, non-irradiated surrogate tissue for the identification of toxicity biomarkers of the systemic agent that are not simultaneously manifesting molecular perturbations elicited by the radiation itself.

### 6.2. Understanding Biological Mechanisms of Toxicity

#### 6.2.1. Biochemical and Functional Markers

Clinical studies which integrate hard endpoints such as observations of adverse responses with gene or protein expression profiles from patient tissues may eventually enhance our understanding of the molecular mechanisms of treatment toxicity. However, so far, relatively little *in vivo* mechanistic data has been available at the patient level, which calls for bridging of molecular omics and clinical phenomics data.

In daily oncology practice, detection of specific clinical organ toxicity is typically done by measuring serum or plasma biochemical markers, such as the determination of liver enzymes and functional factors (e.g., bilirubin and albumin), electrolytes, and blood cell counts. Functional organ testing, such as heart assessment by echocardiography, represents an example of more extensive diagnostic procedures in common use. The analysis of circulating proteins that reflect the grade of treatment-induced enteropathy, if a reliable analytical procedure could be established, would add to the biochemical repertoire that is feasible in routine diagnostics.

#### 6.2.2. Pharmacogenetics

Germline (*i.e.*, heritable) gene variations found among individuals have been utilized as predictive biomarkers of susceptibility for untoward effects to exposure of basically any external agent and ideally, the individualization of treatment protocols [25]. In pharmacogenetics, DNA sequence variants (single-nucleotide polymorphisms) may explain variations in drug absorption, distribution, and excretion, as well as drug targets and downstream effect mediators in normal tissues. For a limited number of systemic cytotoxic agents (*i.e.*, chemotherapeutics) certain variants of germline variations may assist the clinical decision of dose reduction or other modifications in treatment scheduling [43]. However, a recent meta-analysis of studies with fluoropyrimidine-based therapies, the backbone of most CRC chemotherapy regimens, in which comprehensive assessments of toxicities categorized according to CTCAE grading had been undertaken and likewise, numerous genetic biomarkers associated with the specific toxicities had been tested, concluded that the actual test panel did not reach the desired robustness for clinical use [44].

#### 6.2.3. PBMC as Drug-Exposed Normal Tissue

Gene expression profiling of PBMC is commonly used as a correlative analytical strategy for identifying new biomarkers in intervention studies. This normal tissue is readily accessible and can be repeatedly collected in sufficient quantities. Alterations in PBMC gene expression have been shown to reflect responses to a wide range of physiological and pathological conditions, such as dietary modifications, exercise, acute psychological stress, and depression [45,46,47]. It has even been contended that PBMC gene expression profiles may predict therapeutic outcome [45]. Following the initial single-agent vorinostat trial, in which PBMC were analyzed in the quest of biomarkers of HDAC inhibitor activity [48], many other clinical studies of HDAC inhibitors have also applied this strategy.

#### 6.2.4. Phenotypic Validation

Ideally, any new toxicity biomarker will require phenotypic validation. With regard to expected readout data from the LARC-RRP and PRAVO studies, we are currently setting up a validation program using the *C. elegans* model system. This multiorgan animal shares major intracellular and intercellular control pathways with higher organisms; still, its genome is convenient to manipulate and its life cycle is fast. Importantly, tissue responses to DNA damage are remarkably conserved through the evolution. For example, *C. elegans* DNA repair proteins respond to DNA damage by instigating a survival program closely resembling that in human tissues [49,50,51]. Moreover, recent investigations have identified novel *C. elegans* proteins counteracting cytotoxic effects both of fluoropyrimidine and HDAC inhibitor agents [52,53,54]. In the planned validation experiments, we intend to use molecular imaging, reverse and forward genetics, and chemical genetics in functional assays for relevant endpoints (apoptosis, necrosis, heat-shock sensitivity, and others that apply). We envisage this approach to be a rational shortcut to narrow down the number of identified toxicity biomarkers to those of genuine biological cause.

The recent development of human microphysiological systems provides novel opportunities to address challenges faced in predictive toxicity assessment of new systemic compounds. This effort takes a multidisciplinary approach of combining expertise in stem cell biology, material sciences, and bioengineering to build microscale “on-a-chip” models of human multiorgan systems. In such experimental setups, the biologists utilize human primary or stem cell sources which are sustainable over a 4-week period and functionally represent all of the major organ systems upon full differentiation [55]. The engineers employ micro- or nanofabrication technology, which basically provides multicompartment culture chambers connected by fluidic channels, to enable the growth, differentiation, and connection of a few or multiple tissue cultures [56,57]. The potential of such 3-dimensional organ platforms in prediction of tissue perturbations to external stimuli seems promising but still needs developmental experimentation.

It should not be forgotten, in the era of innovative technologies and the resulting new opportunities, that designated model systems and functional bioassays are still highly valuable and above all, feasible tools for phenotypic validation. Validation of organ-typical toxicity features may in their most simplistic form be undertaken in cell monocultures. For example, using cultured normal intestinal epithelial cells, we have mimicked the intestinal toxicity reported by PRAVO study patients [19,21] by the induction of a cell type-specific apoptotic response [58].

### 6.3. Revealing Information Connectivity through Open Data Accessibility

In order to achieve the quested standard of integration of all existing data, which reside in widely located sources within the industrial, academic, regulatory, and clinical practice sectors, an increasing number of scientists are calling for the development of open-source data repositories that allow investigators to access the large and complex data-sets associated with multidimensional analyses with the aim of facilitating the formation of global networks in toxicology and related biological disciplines [59,60]. As templates for future large collaborative studies, such major tool-sets for data mining across different technology platforms will undoubtedly be beneficial as a system to support collaborative projects in general, and particularly as public resources of highly curated data to advance the field of patient safety in cancer therapy [35,61].

## 7. Conclusions

In modern oncology, a number of systemic therapies may have the potential to synergize with DNA-damaging treatment such as radiotherapy. New insights into molecular radiobiology have enabled the design of next-generation trials that formally examine the therapeutic benefit of adding systemic compounds to radiation along with the critically important assessment of adverse effects to the combined-modality treatment. In considering the use of radiosensitizing agents, this may also include the implementation of multiplex high-throughput technologies in the identification of biomarkers as surrogate endpoints of treatment tolerability, which needs particular attention on patient eligibility.

However, mechanism-based prediction of treatment toxicity is a new research avenue in oncology, and extrapolating this field to the discipline of combined-modality cancer therapy adds another layer of complexity. The development of biomarker-driven studies may be a significant challenge in radiotherapy trial design, particularly given the multiplicity of molecular targets that are altered by radiation. There is an additional challenge in the prediction of potential interactions between systemic agents and radiotherapy in patients requiring concomitant or sequential treatment. We are already beginning to see this in routine clinical practice, particularly in the context of patients with advanced cancer, where systemic drugs have enabled longer periods of disease stabilization and also altered pattern of failure, creating new clinical scenarios of patients requiring palliative radiotherapy at the time of co-administration of a systemic drug. In many of these situations, the potential of normal tissue toxicity is unclear.

One of the most controversial issues in studies of this type may be the challenge of large-scale data acquisition and the potential development of open-source data repositories that allow investigators to access the complex data-sets associated with multidimensional analyses. Additionally, the key underlying data, such as quality-assured clinical annotations, are critically important. Recognizing the opportunity of successful open-source networking approaches to storage and sharing of high-quality biological and clinical data, we envisage that the “top-down” strategy of clinical study conduct proceeding to molecular portrayal of toxicity profiles, as outlined in this communication, may be applicable for evaluation, and ultimately, understanding of the complex endpoint of patient safety in contemporary multimodal cancer therapy.
